# Menstrual cycle rhythmicity: metabolic patterns in healthy women

**DOI:** 10.1038/s41598-018-32647-0

**Published:** 2018-10-01

**Authors:** C. F. Draper, K. Duisters, B. Weger, A. Chakrabarti, A. C. Harms, L. Brennan, T. Hankemeier, L. Goulet, T. Konz, F. P. Martin, S. Moco, J. van der Greef

**Affiliations:** 1 Nestle Institute of Health Sciences (NIHS), Lausanne, Switzerland; 2 Mathematical Institute, Leiden University, Leiden, The Netherlands; 3 Division of Analytical Biosciences, Leiden Academic Center for Drug Research, Leiden University, Leiden, The Netherlands; 4 Netherlands Metabolomics Centre, Leiden, The Netherlands; 5 University College Dublin, School of Agriculture and Food Science, Belfield, Dublin 4 Ireland

## Abstract

The menstrual cycle is an essential life rhythm governed by interacting levels of progesterone, estradiol, follicular stimulating, and luteinizing hormones. To study metabolic changes, biofluids were collected at four timepoints in the menstrual cycle from 34 healthy, premenopausal women. Serum hormones, urinary luteinizing hormone and self-reported menstrual cycle timing were used for a 5-phase cycle classification. Plasma and urine were analyzed using LC-MS and GC-MS for metabolomics and lipidomics; serum for clinical chemistries; and plasma for B vitamins using HPLC-FLD. Of 397 metabolites and micronutrients tested, 208 were significantly (p < 0.05) changed and 71 reached the FDR 0.20 threshold showing rhythmicity in neurotransmitter precursors, glutathione metabolism, the urea cycle, 4-pyridoxic acid, and 25-OH vitamin D. In total, 39 amino acids and derivatives and 18 lipid species decreased (FDR < 0.20) in the luteal phase, possibly indicative of an anabolic state during the progesterone peak and recovery during menstruation and the follicular phase. The reduced metabolite levels observed may represent a time of vulnerability to hormone related health issues such as PMS and PMDD, in the setting of a healthy, rhythmic state. These results provide a foundation for further research on cyclic differences in nutrient-related metabolites and may form the basis of novel nutrition strategies for women.

## Introduction

The monthly menstrual cycle represents one of many physiological rhythms essential for life. The heartbeat and daily sleep-wake cycle represent obvious rhythms. Less obvious are the physiological processes inside the body such as the rhythmicity of the sex hormones that drive the menstrual cycle and others that regulate growth and metabolism^1^. These rhythms also interact with each other through synchronization of cellular activities with the external environment through feedback mechanisms that promote dynamic stability, such as the interaction between circadian rhythms, sleep and the menstrual cycle^2,3^. Perturbations of the body’s rhythmic processes are associated with disorders^4^ such as disturbed circadian rhythmicity with premenstrual dysphoric disorder (PMDD)^5^ or abnormal expression of the circadian clock gene and spontaneous abortion^6^.

**Figure 1:**
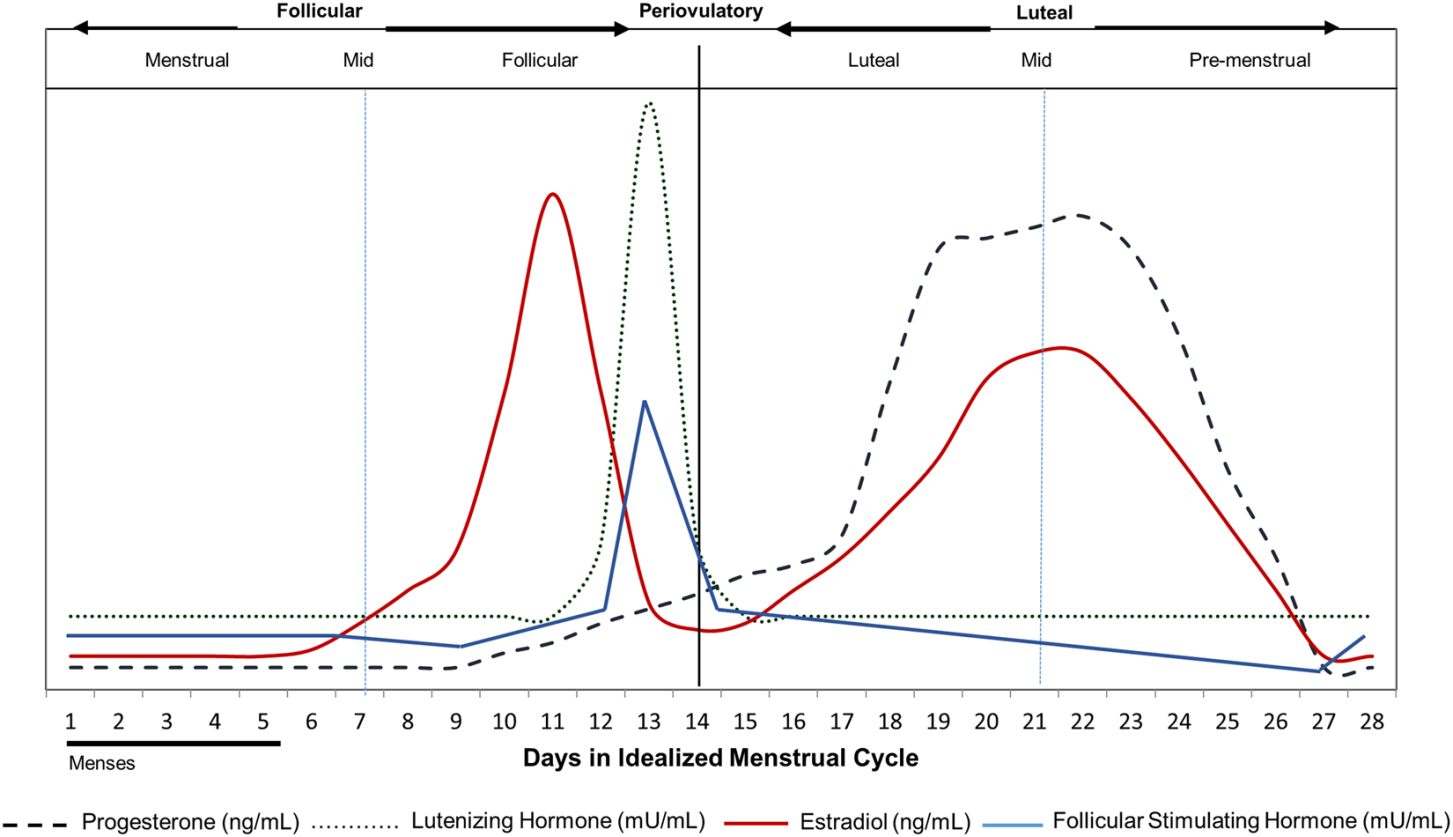
Hormone levels according to menstrual cycle phase. Changing concentrations of female sex hormones (progesterone, luteinizing hormone, follicular stimulating hormone, estradiol) that characterize the 5 phases (menstrual, follicular, periovulatory, luteal and pre-menstrual) of the menstrual cycle (adapted with permission^7^). Follicular stimulating hormone concentration changes overlayed^9^.

The first half of the menstrual cycle is comprised by the menstrual and follicular phases during which time estrogen levels are low (menstrual phase) and rise (follicular phase) and ends with the periovulatory phase in which follicular stimulating hormone (FSH) and luteinizing hormones (LH) peak. The second half of the cycle is comprised by the luteal (during which time estrogen level rises with a progesterone peak) and the pre-menstrual phases during which time estrogen and progesterone levels fall (Fig. 1)^7–9^). However, it is during this time that women experience worsening of chronic diseases such as diabetes and inflammatory bowel disease, bloating, poor sleep quality, and premenstrual syndrome (PMS) or PMDD^10–18^. Moreover, the luteal phase is also accompanied by decreasing amino acid levels and elevated nitrogen utilization^19,20^. Women with PMS and PMDD have an increased appetite, food cravings and excess calorie intake which are associated with cyclical changes in serotonin during this period^12,21–23^. These biochemical changes suggest nutrient utilization is affected by changing sex hormones between phases. The luteal phase of the menstrual cycle may be considered a normally stressed physiology which amplifies differential responses between individuals to environmental stressors such as diet intake. These differential responses might predict future chronic health issues.

**Figure 2:**
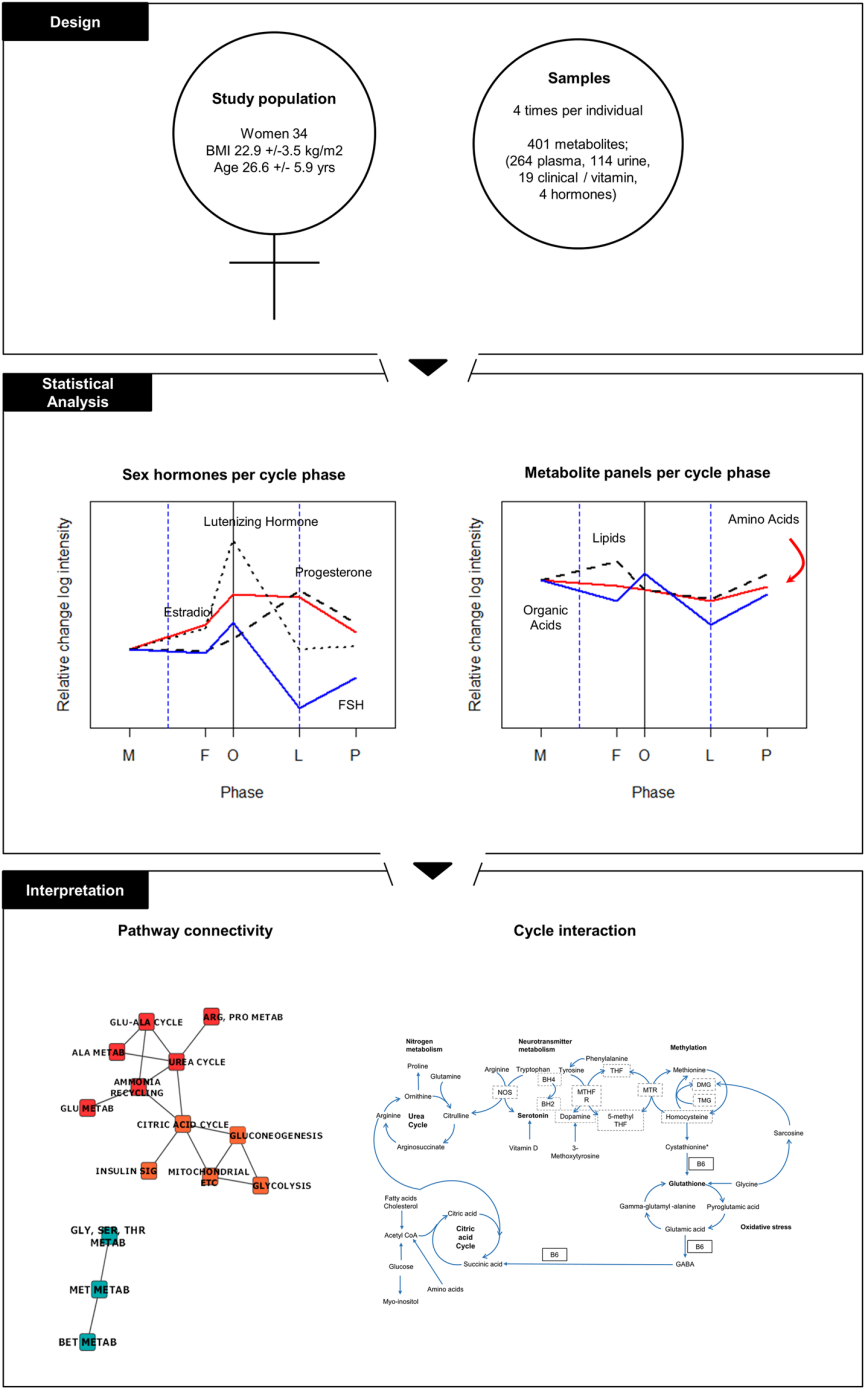
Study schema. Thirty-four women (BMI 22.9 +/− 3.5 kg/m^2^, age 26.6 +/− 5.9 yrs) provided 4 blood and urine samples that each uniquely fit into 1 of 5 phase timepoints based on 4 sex hormone measurements (LH, FS, estradiol, and progesterone) and self-reported menstrual cycle timing. A total of 401 metabolites were measured which included 263 plasma, 114 urine, and 19 clinical and vitamin analyses. Metabolite profiling was conducted and statistically significant rhythmicity is depicted for the amino acid, lipid and organic acid panels. Biochemical pathway interconnectivity was identified between the urea cycle, 1 carbon metabolism, glutathione metabolism and the citric acid cycle. M-menstrual, F-follicular, O-Periovulatory, L-luteal, P-premenstrual phases.

In order to characterize baseline metabolic rhythmicity in the menstrual cycle, advanced metabolomic profiling, clinical and nutrient biochemistries were analyzed for rhythmic variations throughout a healthy menstrual cycle in 34 healthy women. Samples were analysed from five different phases of the menstrual cycle obtained from a previously published study^19^. The results are interpreted in light of metabolic differences that may represent vulnerability to sex hormone related disorders and nutritional needs, as well as diagnostic and therapeutic approaches that vary across the menstrual cycle (Fig. 2).

## Results

Analysis of the plasma levels of estradiol, progesterone, luteal hormone and follicular stimulating hormone followed the expected temporal concentration profiles of cycle phases, however, the estradiol concentration in the follicular phase was lower than the luteal phase peak (Supplementary Fig. 1 vs. Fig. 1). This variation may represent the small number of women in a narrow age range in this study.

The 4 sampling windows of the original study were timed to capture the menstrual (M), follicular (F), periovular (O), luteal (L) and pre-menstrual (P) phases of the cycle. All participants were observed during one monthly cycle. A total of 33, 31, 15, 27, and 11 samples were available for the M,F,O,L,P phases respectively; and 117 samples were available for analysis of all metabolites (Supplementary Table 1).

**Figure 3:**
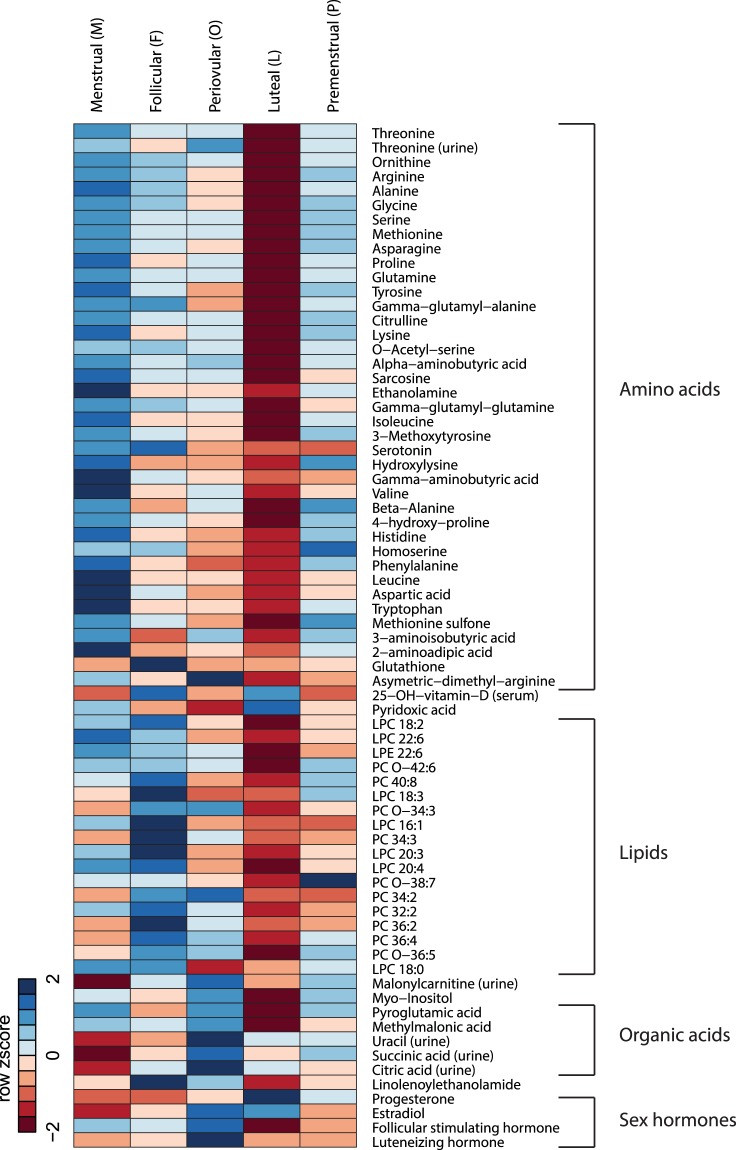
Metabolites vary across menstrual cycle phase. This heatmap with color gradients indicates rhythmicity across the menstrual cycle. Lower amino acid and lipid metabolite concentrations are visualized in the luteal phase. Phase means of logarithmically transformed metabolite data are row standardized in the heatmap to obtain Z scores. Two cells that are close in color represent similar Z scores, ranging from blue (Z equals minus 2) to red (Z equals plus 2). Amino acid, lipid, organic acid and sex hormone variables are ordered according to main biochemical pathways or classes and depicted at q < 0.20 after contrast analyses. Menstrual (M), Follicular (F), Periovulatory (O), Luteal (L), Premenstrual (P) phases are depicted. LPC- Lysophosphatidylcholine, LPE- Lysophosphatidylethanolamine, PC-phosphatidylcholine.

**Figure 4:**
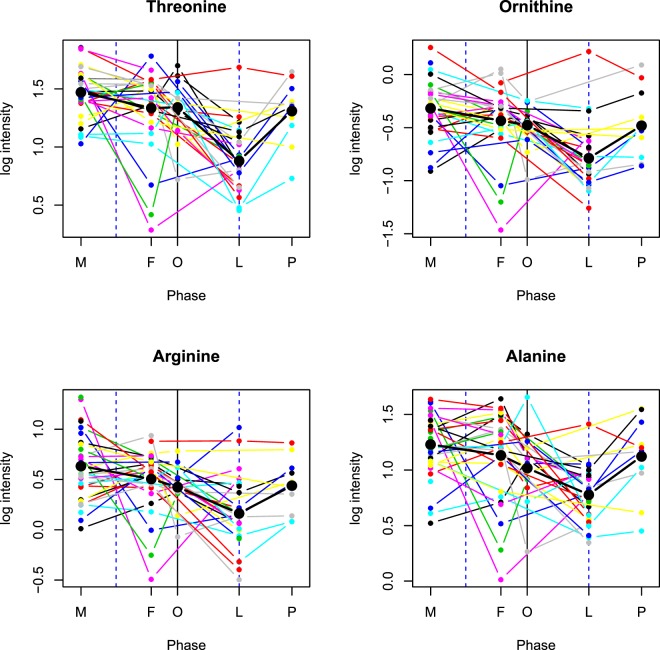
Amino acid variability by cycle phase. Mean log intensity is depicted along with individual variability for threonine, ornithine, arginine, alanine, glycine, serine, methionine, asparagine, proline, glutamine, tyrosine, gamma-glutamyl-alanine, citrulline, o-acetyl-serine, alpha-aminobutyric acid, and gamma-glutamylglutamine at one time point for each of the 5 menstrual phases (M = menstrual, F = follicular, O = periovular, L = luteal, p = premenstrual). Each colored line represents an individual. Amino acids are depicted which have 2 or more contrast comparisons meeting the multiple testing threshold of q < 0.20. Statistically significant luteal phase reductions can be observed.

Since the overall research goal was to investigate menstrual cycle metabolic rhythmicity, it is natural to assess phase dynamics of individual biochemical species. To do so, calculated phase means of each biochemical species were compared, while taking the participant-specific nature of the data into account. Each phase-phase difference, or contrast, is tested for statistical significance before (p < 0.05) and after multiple testing control of the false discovery rate (q < 0.20; details in the Methods section). Logarithmically transformed statistically significant metabolite patterns, per cycle phase, meeting q value threshold q < 0.20 can be visualized in Fig. 3. Mean log intensity and individual variability of metabolites with 2 or more statistically significant contrast comparisons meeting q < 0.20 are depicted in Fig. 4.

### Plasma amino acids and biogenic amines were significantly lower in the luteal phase

Out of the 54 amino acids and derivatives and biogenic amines detected, 48 reached statistical significance (p-value < 0.05) in the 5 phase contrast comparisons: luteal-follicular (L-F), luteal-menstrual (L-M), luteal-periovulatory (L-O), premenstrual-luteal (P-L) and peri-ovular-menstrual (O-M) (Table 1 and Supplementary Table 1).

**Table 1:** Measured sex hormone (progesterone, estradiol, follicular stimulating hormone, lutenizing hormone) and 39 amino acid effect sizes and q values are listed for all significant phase comparison categories.

Biomarkers vs. Phase contrasts	Effect Size**					FDR q****				
	L-F*	L-M	L-O	P-L	O-M	q L-F	q L-M	q L-O	q P-L	q O-M
**Sex Hormones**, **serum**										
Progesterone (ng/L)	2.88	2.80	2.30	−1.51	0.50	0	0	0	**2**.**00E-11**	**7**.**87E-02**
Estradiol (pmol/L)	0.94	1.79	−0.10	−1.19	1.89	**1**.**32E-09**	0		**2**.**88E-08**	0
Follicular stimulating hormone (FSH) (IU/L)	−0.75	−0.80	−1.16	0.42	0.36	0	0	0	**1**.**37E-03**	**3**.**52E-03**
Lutenizing hormone (LH) (IU/L)	−0.27	0.00	−1.40	0.04	1.39	2.72E-01		0		0
**Amines**, **plasma**										
Threonine	−0.45	−0.59	−0.46	0.43	−0.13	**6**.**73E-09**	0	**2**.**24E-05**	**1**.**67E-03**	
Ornithine	−0.35	−0.47	−0.31	0.31	−0.16	**2**.**12E-05**	**2**.**10E-11**	**4**.**67E-02**	**9**.**32E-02**	
Arginine	−0.34	−0.47	−0.26	0.28	−0.21	**5**.**51E-04**	**1**.**87E-08**	3.47E-01	3.62E-01	
Alanine	−0.35	−0.45	−0.24	0.35	−0.21	**3**.**93E-04**	**3**.**29E-08**	4.59E-01	**9**.**32E-02**	
Glycine	−0.31	−0.40	−0.23	0.34	−0.17	**4**.**36E-04**	**6**.**16E-08**	3.71E-01	**9**.**32E-02**	
Serine	−0.26	−0.37	−0.25	0.28	−0.11	**2**.**50E-03**	**9**.**20E-08**	**1**.**93E-01**	**1**.**01E-01**	
Methionine	−0.25	−0.37	−0.22	0.28	−0.15	**4**.**95E-03**	**9**.**20E-08**	3.47E-01	**1**.**01E-01**	
Asparagine	−0.27	−0.37	−0.22	0.30	−0.15	**2**.**89E-03**	**5**.**24E-07**	4.52E-01	**9**.**32E-02**	
Proline	−0.21	−0.38	−0.18	0.29	−0.20	**1**.**30E-01**	**1**.**57E-06**		2.05E-01	
Glutamine	−0.24	−0.32	−0.21	0.24	−0.11	**4**.**21E-03**	**2**.**58E-06**	3.47E-01	**1**.**94E-01**	
Tyrosine	−0.18	−0.32	−0.12	0.25	−0.20	**1**.**74E-01**	**1**.**32E-05**		3.04E-01	
γ-glutamyL-alanine	−0.49	−0.51	−0.23	0.33	−0.28	**3**.**93E-04**	**1**.**34E-05**		7.02E-01	
Citrulline	−0.22	−0.31	−0.23	0.26	−0.09	**2**.**11E-02**	**1**.**34E-05**	3.47E-01	**1**.**87E-01**	
Lysine	−0.18	−0.32	−0.21	0.23	−0.11	2.26E-01	**1**.**34E-05**	4.59E-01	4.63E-01	
O-AcetyL-serine	−0.31	−0.33	−0.25	0.24	−0.07	**5**.**51E-04**	**2**.**12E-05**	2.90E-01	3.82E-01	
α-aminobutyric acid	−0.25	−0.37	−0.32	0.23	−0.05	**4**.**81E-02**	**2**.**33E-05**	**1**.**74E-01**	9.54E-01	
Sarcosine	−0.17	−0.32	−0.09	0.17	−0.23	3.69E-01	**5**.**34E-05**			
Ethanolamine	−0.11	−0.23	−0.07	0.11	−0.16		**4**.**98E-04**			
γ-glutamylglutamine	−0.34	−0.41	−0.28	0.22	−0.13	**3**.**51E-02**	**7**.**35E-04**			
L-Isoleucine	−0.14	−0.24	−0.08	0.16	−0.16		**1**.**09E-03**			
Methoxytyrosine	−0.20	−0.25	−0.16	0.21	−0.09	**7**.**40E-02**	**1**.**17E-03**		6.48E-01	
Seroton	−0.71	−0.57	−0.12	0.06	−0.46	**1**.**17E-03**	**6**.**95E-03**			
Hydroxylysine	−0.08	−0.22	−0.05	0.17	−0.17		**1**.**53E-02**			
γ-aminobutyric acid	−0.10	−0.22	−0.05	0.02	−0.17		**1**.**58E-02**			
Valine	−0.11	−0.21	−0.08	0.11	−0.13		**1**.**66E-02**			
β-Alanine	−0.05	−0.11	−0.06	0.09	−0.05		**1**.**72E-02**			
4-hydroxy-proline	−0.26	−0.32	−0.19	0.35	−0.13	2.42E-01	**1**.**78E-02**			
Histidine	−0.09	−0.19	−0.06	0.14	−0.13		**3**.**89E-02**			
Homoserine	−0.23	−0.24	−0.09	0.34	−0.15	**1**.**61E-01**	**4**.**08E-02**		**1**.**49E-01**	
Phenylalanine	−0.08	−0.19	−0.01	0.14	−0.17		**4**.**79E-02**			
Leucine	−0.07	−0.18	−0.05	0.09	−0.13		**4**.**79E-02**			
Aspartic acid	−0.15	−0.28	−0.07	0.10	−0.21		**1**.**00E-01**			
Tryptophan	−0.08	−0.18	−0.05	0.10	−0.13		**1**.**06E-01**			
Methionine sulfone	−0.14	−0.17	−0.09	0.16	−0.08		**1**.**12E-01**			
3-aminoisobutyric acid	−0.07	−0.19	−0.21	0.11	0.02		**1**.**74E-01**			
2-aminoadipic acid	−0.06	−0.18	−0.05	0.10	−0.14		**1**.**91E-01**			
Glutathione	−0.33	−0.01	0.00	0.01	−0.02	**1**.**61E-01**				
Assymetric-dimethyl-arginine (ADMA)	−0.08	−0.14	−0.22	0.03	0.08		**1**.**96E-01**	2.36E-01		
**Amines, urine**										
Threonine	−0.29	−0.40	−0.54	0.19	0.14		3.54E-01	**1**.**79E-01**		

*Luteal/follicular (L-F), luteal-menstrual (L-M), luteal-periovulatory (L-O), premenstrual-luteal (P-L) and peri-ovular-menstrual (O-M); **All results are based on natural log transformed data. Effect sizes are estimated phase-phase differences (“contrasts”). ***Student’s t-test p-values of the contrasts are controlled for multiple testing within metabolites by Bonferroni and across metabolites using Benjamini-Hochberg FDR, resulting in q values as listed. Significant q values are in bold. Values above 0.50 are intentionally left blank.

Ornithine, arginine, alanine, glycine, methionine, and proline were statistically significant in all 5 phase contrast comparisons, with the luteal phase showing a statistically significant reduction in amines relative to the other phases. After correction for multiple testing and using a q value threshold <0.20, 37 amines reached statistical significance in the L-M contrast. Nineteen of these same amines met the q value threshold <0.20 for the L-F, 4 for L-O and 9 for P-L (Table 2 and Fig. 4). Total glutathione was statistically significant only for L-F. Threonine, ornithine, and serine show significance across the 4 phase contrast comparisons; L-M, L-F, L-O, and P-L (q < 0.20) (Table 1 and Supplementary Table 2).

**Table 2:** Measured vitamins (25-OH-vitamin D, pyridoxic acid),lipids (7 LPC, 1 LPE, 10 PC), malonylcarnitine, plasma organic acids (inositol, pyroglutamic acid, methylmalonic acid), urine organic acids (uracil, succinic acid, citric acid) and linolenylethanolamide are listed for all significant phase comparison categories.

Biomarkers vs. Phase contrasts	Effect Size**					FDR q values***				
	L-F*	L-M	L-O	P-L	O-M	q L-F	q L-M	q L-O	q P-L	q O-M
**Vitamins**										
25-OH-vitamin-D - serum (nmol/L)	−0.06	−0.09	0.01	0.00	−0.10	4.82E-01	1.21E-02			**3.85E-02**
Pyridoxic acid - plasma (nmol/L)	0.05	−0.16	0.29	0.18	−0.45					**1.18E-01**
**Lipids**, **plasma**										
LPC 18:2	−0.26	−0.17	−0.08	0.22	−0.09	**4**.**78E-04**	2.89E-01			
LPC 22:6	−0.20	−0.18	−0.09	0.17	−0.09	**1**.**76E-02**	2.89E-01			
LPE 22:6	−0.34	−0.27	−0.21	0.27	−0.06	**1**.**45E-02**	2.89E-01			
PC O-42:6	−0.13	−0.09	−0.06	0.10	−0.03	**1**.**11E-02**	9.34E-01			
PC 40:8	−0.15	−0.07	−0.01	0.09	−0.06	**2**.**41E-03**				
LPC 18:3	−0.30	−0.09	0.02	0.26	−0.11	**1**.**76E-02**				
PC O-34:3	−0.14	−0.02	−0.09	0.09	0.08	**2**.**64E-02**				
LPC 16:1	−0.20	−0.11	−0.02	0.05	−0.08	**1**.**76E-02**				
PC 34:3	−0.19	−0.02	−0.04	0.10	0.02	**3**.**30E-02**				
LPC 20:3	−0.16	−0.07	0.02	0.07	−0.10	**3**.**83E-02**				
LPC 20:4	−0.17	−0.12	−0.06	0.12	−0.06	**1**.**76E-02**				
PC O-38:7	−0.09	−0.02	−0.04	0.11	0.02	**7**.**74E-02**				
PC 34:2	−0.08	−0.02	−0.06	0.02	0.03	**3**.**30E-02**				
PC 32:2	−0.26	−0.19	−0.05	0.17	−0.14	**3**.**30E-02**				
PC 36:2	−0.09	−0.02	−0.02	0.03	0.00	**9**.**41E-02**				
PC 36:4	−0.08	−0.01	−0.04	0.03	0.02	**1**.**26E-01**				
PC O-36:5	−0.13	−0.03	−0.07	0.10	0.04	**1**.**26E-01**				
LPC 18:0	−0.11	−0.11	0.08	0.03	−0.19	4.78E-01				**1**.**60E-01**
**Acylcarnitines**, **urine**										
Malonylcarnitine	−0.18	0.18	−0.43	0.19	0.61					**1**.**96E-01**
**Organic Acids**, **plasma**										
Inositol	−0.20	−0.30	−0.40	0.30	0.10		**9**.**53E-02**	**9**.**62E-02**	1	1
Pyroglutamic acid	−0.14	−0.39	−0.46	0.23	0.07		**2**.**11E-02**	**9**.**62E-02**		
Methylmalonic acid	−0.12	−0.18	−0.13	0.06	−0.04		**9**.**53E-02**			
**Organic Acids**, **urine**										
Uracil	0.27	0.39	−0.23	−0.28	0.61		4.11E-01			**3**.**74E-02**
Succinic acid	−0.03	0.26	−0.30	0.07	0.57					**9**.**67E-02**
Citric acid	0.00	0.17	−0.16	−0.03	0.33					**1**.**80E-01**
**Endocannabinoids**, **plasma**										
Linolenoylethanolamide (LEA)	−0.15	−0.08	−0.11	0.02	0.03	**1**.**92E-02**				

*Luteal/follicular (L-F), luteal-menstrual (L-M), luteal-periovulatory (L-O), premenstrual-luteal (P-L) and peri-ovular-menstrual (O-M); **All results are based on natural log transformed data. Effect sizes are estimated phase-phase differences (“contrasts”). ***Student’s t-test p-values of the contrasts are controlled for multiple testing within metabolites by Bonferroni and across metabolites using Benjamini-Hochberg FDR, resulting in q values as listed. Significant q values are in bold.Values above 0.50 are intentionally left blank. LPC, Lysophosphatidylcholine, LPE- Lysophosphatidylethanolamine, PC-phosphatidylcholine.

Analysis of the same amino acids and biogenic amines in urine yielded data for 60 compounds. Twenty amino acids were statistically significant (p < 0.05, Table 1) between phases for at least 1 phase contrast (L-F, L-M, L-O, O-M) with L-O and O-M having the highest number of statistically significant differences (Table 1 and Supplementary Table 1).Threonine differed in 3 comparisons and was the only amino acid that reached the FDR threshold in urine (q < 0.20 for L-O).

### Plasma phospholipids were significantly reduced in the luteal phase

Of the 139 lipid species with detectable plasma levels, 57 reached statistical significance, (p-value < 0.05), for 1 to 5 phase contrast comparisons: L-F, L-M, L-O, P-L and O-M (specific p-values in Table 2). Thirty eight percent of the lipid species tested (53/139) consistently showed a statistically significant decrease in the luteal phase relative to the follicular and in some cases, relative to the menstrual phase (16/139) with 7 compounds showing a decrease in comparison to the premenstrual phase and 2 in comparison to the periovulatory phase. One compound, LPE 22:6, showed a statistically significant difference in 4 out of the 5 phase contrasts: L-F, L-M, L-O and P-L. After multiple testing, at q < 0.20, 17 lipid species met this threshold for L-F including 6 LPCs, 10 PCs and 1 LPE. One other LPC met this threshold for O-M (Table 2 and Supplementary Table 2).

### Vitamin D and pyridoxic acid increased in the menstrual phase

Nineteen clinical parameters were tested including eight B vitamins, cofactors and metabolites. C reactive protein (CRP) was statistically significant in L-F (p < 0.05), while high density lipoprotein (HDL), triglycerides and cholesterol were statistically significant in L-F (p < 0.05). Glucose showed rhythmicity with a statistically significant decrease in the luteal phase in comparison to menstrual, pre-menstrual and periovulatory phases (p < 0.05). Magnesium showed a statistically significant decrease in L-M and O-M and riboflavin showed a statistically significant decrease in luteal vs. pre-menstrual phases. However, these results were not significant when corrected for multiple testing (q < 0.20). Vitamin D (25-OH vitamin D) showed significant decreases in L-F, L-M and O-M with L-M and O-M and met the multiple testing threshold q < 0.20). Moreover, the menstrual phase consistently showed higher levels of vitamin D. Pyridoxic acid also showed an elevation in the menstrual compared to the periovulatory phases (q < 0.20, Table 2 and Supplementary Table 2).

### Plasma and urine acylcarnitines showed a trend towards an increase in the periovulatory phase

Of the 50 compounds tested in the acylcarnitine panel, 19 were statistically significant (Table 2) in plasma and 16 statistically significant in urine (p < 0.05) (Table 2). The majority of plasma and urine metabolites were altered in O-M with an increase in the periovulatory phase. However, only urinary malonylcarnitine reached the q < 0.20 threshold for multiple testing (Table 2 and Supplementary Table 2).

### Organic acids and endocannobinoids showed different patterns between phases

Sixteen organic acid metabolites had concentration levels above the respective limit of quantification in plasma out of which 10 reached statistical significance, p < 0.05. Inositol, pyroglutamic acid and methylmalonic acid reached the multiple testing threshold (q < 0.20) for L-M for all 3 metabolites and L-O for inositol and pyroglutamic acid.

Twenty-three organic acid metabolites were analyzed in urine, of which 14 were statistically significant, p < 0.05, in various contrasts. Uracil, succinic acid and citric acid reached the multiple testing threshold q < 0.20 for O-M (Table 2 and Supplementary Table 2).

In plasma, 19 endocannabinoids had detectable levels, of which 5 demonstrated statistical significance p < 0.05 across L-F, L-M, L-O, and O-M; and LEA reached statistical significance for L-F,L-M and L-O and met the multiple testing threshold (q < 0.20) for L-F (Table 2 and Supplementary Table 2).

### Metabolite reactions and subsystems analyses demonstrated interconnectivity and differentiation between menstrual cycle phases

The KEGG database and Human RECON model were used to enrich functional understanding of the 71 compounds that met the multiple testing q-value (<0.20). KEGG currently maps 18,111 metabolites to 519 pathways and RECON 2.2 uses 5324 metabolites and 7785 primarlly intracelluar reactions. Sixty-two of the 71 compounds in this study were identified in the KEGG pathways and 41 of these 62 entitites (Supplementary Table 3) could be mapped in RECON 2. These 41 metabolites participate in 1213 reactions (out of the 7440 reactions tabulated). We do not consider the 710 reactions for extracellular transport and the 84 reactions for exchange/demand. Thus, 419 reactions remained for further analysis and interpretation. Metabolite subsystem analyses of the 41 metabolic entities in the 419 reactions identified showed reduced glutathione, succinate, L-histidine and glycine followed by L-lysine, L-alanine, L-arginine and L-serine impact or are impacted by the most number of metabolic reactions (Supplementary Fig. 2A).

The 34 impacted sub-systems in the metabolic landscape include amino acid metabolism/synthesis; such as glutathione metabolism and the urea cycle; eicosanoid metabolism; citric acid cycle and bile acid synthesis. (Supplementary Fig. 2b) show the global, significant impact of changes in menstrual phase on metabolism.

A deeper pathway analysis in which interconnected, amino acid phase contrasts were compared, revealed minor differences between the 2 significant phase contrast categories. The luteal menstrual contrast did not show a significant difference in glutathione levels like the luteal follicular contrast (Supplementary Fig. 3).

## Discussion

The present study demonstrates the rhythmic synchronicity of the menstrual cycle with healthy metabolism. Using deep molecular phenotyping of 5 menstrual cycle phases, paired with sex hormone rhythmicity, 67 biochemical species of amino acid, lipid, carbohydrate, energy and vitamin metabolism significantly changed between phases; particularly, with a decrease in the luteal relative to menstrual and follicular phases (Fig. 3). Much fewer changes were observed in the urine in comparison to the plasma. These biochemical species comprise major biochemical pathways which impact physiological functioning and may increase vulnerability to sex-hormone related disorders, such as PMS, PMDD, and polycystic ovarian syndrome (PCOS).

**Figure 5:**
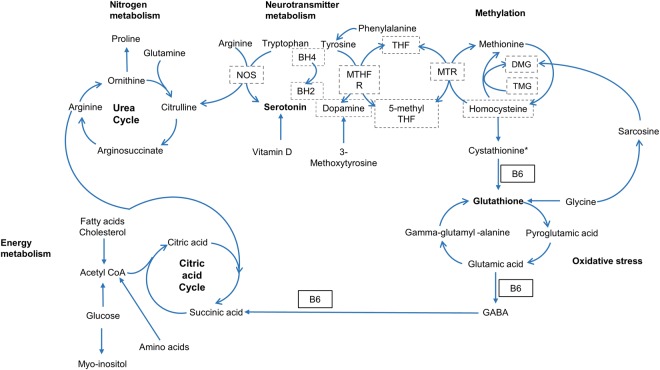
Rhythmic metabolites in the urea cycle, neurotransmitter metabolism connect with 1 carbon, glutathione metabolism and the citric acid cycle. The metabolites with FDR controlled rhythmicity participate in inter-related, biochemical pathways including nitrogen metabolism (the urea cycle), neurotransmitter metabolism, methylation (1 carbon metabolism), oxidative stress (glutathione metabolism) and energy metabolism (citric acid cycle). NOS = Nitric oxide synthase; BH4 = Tetrahydrobiopterin; BH2 = Bihydrobiopterin; MTHFR = Methylenetetrahydrofolate reductase; THF = Tetrahydrofolate; MTR = Methionine synthase; DMG = Dimethylglycine; TMG = Trimethylglycine; B6 = Vitamin B6. Compounds boxed with dotted lines (NOS, BH4, BH2, MTHFR, THF, MTR, 5-methyl THF, DMG, TMG, homocysteine) were not evaluated or not significant (dopamine). All metabolites without dotted lines met the multiple testing threshold q < 0.20. *Cystathionine was statistically significant with p value < 0.05, but did not meet the multiple testing threshold.

Luteal phase protein, lipid, steroid, endometrial biosynthesis and increased energy utilization may lead to reduced biomarkers relative to menstrual and follicular phases. The decrease in amino acid plasma levels observed in the luteal phase, particularly in comparison to the menstrual phase, may be associated with progesterone’s upregulation of cell cycle progression and growth and the associated protein biosynthesis required for endometrial thickening to prepare the uterus for pregnancy^8^ (Table 1 and Figs 3 and 4). The decreased amino acid concentrations that participate in the urea cycle (arginine, ornithine and citrulline) suggest reduced ammonia waste in the luteal phase, which supports progesterone’s anabolic amino acid use (Fig. 5). Sex hormone regulation of nitrogen utilization through nitrogen excretion fluctuation^20,24^ and reduced concentrations of amino acids in the luteal phase suggest the intake of a higher protein load might, in certain instances, be advantageous to support additional nitrogen needs. Women have higher energy expenditure and compensate by eating more in the luteal phase, particularly protein; suggesting the anabolism in this phase could be greater than the degree of difference we observe using the tested metabolomics technologies^25,26^.

The anabolic effect of the luteal phase does not appear to be limited to amino acids, as certain lipids decreased in the luteal relative to follicular phases suggesting a higher utilization of fat for lipid or steroid synthesis, and/or an increase in fat absorption with less need for anabolism in the follicular phase (Table 2 and Fig. 3). Previous research demonstrates total phospholipid content of the endometrium is increased in the luteal phase by 26% relative to the periovulatory time-period^27^. A 10-fold increase of phospholipase A_2_ in endometrial tissue has previously been identified in the luteal phase^28^. Partial hydrolysis of phosphatidylcholines (PC) and phosphatidylethanolamines (PE) by phospholipase A_2_ produces lysophosphatidlycholines (LPC) and lysophosphatidlyethanolamines (LPE). PE and LPC are minor phospholipids found in cell membranes such as myelin sheaths and erythrocytes; playing roles in cell signaling and enzyme activation. Our findings of decreased PCs, LPCs, PEs and LPEs are consistent with their anabolic use for endometrial tissue thickening for pregnancy preparation during the luteal phase. Previous research identified PCs, LPCs and LPEs are further reduced in the luteal phase of PCOS patients versus healthy controls^29,30^. Thus, lower phospholipids in the luteal phase could be physiologically normal. However, certain diseases may perturb this state further, suggesting augmentation of physiologic vulnerability and highlighting the importance of studying hormonal rhythmicity in health and disease.

Endocannabinoids are known to interact with sex hormones and cytokines to regulate fertility^31^. Conversely, changes in levels of sex hormones are known to alter endocannabinoid signaling^32^. The central nervous system is a rich source of endocannabinoids, highly sensitive to inflammation and this interaction is implicated in PMDD^33^. Additionally, endocannabinoids are generated from membrane phospholipids^31,34^. In the present study, one endocannabinoid, linolenoyl ethanolamide (LEA), demonstrated luteal phase rhythmicity, which has not been demonstrated previously (Table 2). The low concentration of LEA observed may be a result of phosphotidylethanolamine use for endometrium development. This results in less LEA available for endocannabinoid generation. Further research is needed to identify if this can increase vulnerability to a sub-optimal stress response in individuals susceptible to PMS or PMDD, particularly when combined with low concentrations of amino acid precursors of neurotransmitter metabolism.

Medium and longchain acylcarnitines are formed from fatty acid oxidation, and elevated in inflammation, menopause and lower in PCOS^35,36^. In our healthy, premenopausal population, a trend of upregulated acylcarnitines was observed in the periovular phase from urine and plasma which may reflect a higher state of inflammation and demand for beta oxidation and energy utilization^37^ (Supplementary Table 2).

Clinical laboratory diagnostics used in practice can vary with the menstrual cycle due to increased anabolic demands in the luteal phase, for example, and should be interpreted with caution. Cholesterol is a key constituent of sex hormones and is utilized during the luteal phase for progesterone and estrogen synthesis. In the present study, cholesterol and HDL showed significant trends with reductions in the luteal relative to the follicular phases and, consistent with prior literature^38,39^. Triglyceride concentrations are known to be reduced in the luteal phase, as was observed in our study; and, more specifically, are reduced 30% from estradiol treatment (but not progesterone) due to accelerated VLDL-TG plasma clearance^40,41^. Thus, caution is warranted when interpreting cholesterol and triglyceride laboratory results (Table 2).

While not yet accepted as a biomarker for clinical practice, inositol is produced by the human body from glucose and may be in high demand to meet the anabolic requirement of luteal phase pregnancy preparation. It plays a key role in insulin signal transduction, lipid transport and catabolism, oocyte maturation, embryonic development and cytoskeleton assembly which influences the steroidogenesis process^42–44^. We observed a significant reduction in luteal phase myo-inositol (Table 2). The trend observed of reduced glucose concentration in the luteal phase may have led to the reduction in inositol production (Supplementary Table 2).

Significant neurotransmitter, amino acid and B vitamin precursor rhythmicity may influence susceptibility to the cyclical stress, anxiety and depression implicated in PMS and PMDD. Women are more affected than men by depressive disorders during the time between menarche and menopause suggesting this increase in depression risk is sex hormone related^45–47^. For example, γ-amino-butyric-acid (GABA) inhibition has been implicated in depression pathophysiology, differs between men and women, and can be modulated by progesterone and estrogen^48,49^. We have observed a significant reduction of the neurotransmitter serotonin and several neurotransmitter metabolic precursors (tyrosine, tryptophan, 3-methoxytyrosine, GABA, L-phenylalanine) which is consistent with previous research that demonstrates reduced mood enhancing neurotransmitter metabolite levels in the luteal phase, such as, 5-hydroxyindoleacetic acid (5-HIAA), the serotonin metabolite^50^ (Table 1, and Fig. 5).

Four-pyridoxic acid is one of two major vitamin B-6 compounds present in plasma. It is a cofactor in sex hormone gene expression and neurotransmitter metabolism through the conversion of tryptophan to serotonin^51^. It is also a cofactor for GABA synthesis (Fig. 5). Supplementation with B6 has been shown to improve the psychiatric symptoms of PMS^52,53^. In our study, 4-pyridoxic acid was significantly lower in the periovulatory phase, which may increase susceptibility to premenstrual syndrome in vulnerable individuals should there be insufficient B6 for tryptophan to serotonin conversion in the brain (Table 2). The periovulatory reduction in 4-pyridoxic acid may lead to the cystathionine depletion observed in the luteal phase as B6 is a cofactor for the cystathione-β−synthase conversion to cystationine (Table 2 and Fig. 5).

Rhythmicity in glutathione and associated metabolites may lead to oxidative stress and impaired liver detoxification and may be associated with sex hormone influences in oxidative stress and drug metabolism^54–56^. Sex hormones have been shown to be correlated with redox balance during the menstrual cycle in the endometrium through modulation of glutathione metabolism^57^. In the present study, plasma total glutathione and its’ precursors; glycine, γ- glutamyl-alanine, and pyroglutamic acid showed significant differences across the menstrual cycle with the precursors following the same pattern of lowest concentrations in the luteal phase (Table 1 and Fig. 5). In the present study, the reduced glutathione precursors in the luteal phase significantly increased in the menstrual phase, which may be necessary to precede glutathione’s successful follicular phase regeneration (Supplementary Fig. 3). Previous findings correlate elevated glutathione and glutathione peroxidase activity with the estrogen peak in the follicular phase^58–60^; and may be one mechanism through which estrogens attenuate oxidative stress^61,62^. Individuals with PMS have been shown to have an imbalance in oxidant/antioxidant status and may be more susceptible in a state of low glutathione metabolic activity in the luteal phase^63^.

Vitamin D supplementation, when combined with calcium, has been used to improve weight loss, menstrual regularity, hyperandrogenism and, possibly, fertility; in women with PCOS^64^. High dietary intake of vitamin D may reduce risk of PMS which may be related to its capacity to activate serotonin synthesis^65,66^. Vitamin D regulates calcium and bone health, sex steroidogenesis, and interacts with progesterone to regulate the immune system through T cell induction of the vitamin D receptor^67^. A reduction of 25-hydroxyvitamin D (25OH-vitamin D) associated with a decrease in estradiol is seen in post-menopause and likely related to the sensitive interdependency between changes in estrogen levels and vitamin D binding protein^68^. In human follicular cells, it alters FSH sensitivity and participates in folliculogenesis and progesterone production, indicating a possible role in follicular development and luteinization^69^. In our study, the significant reduction of vitamin D in the luteal and periovulatory phases may reflect a higher utilization for folliculogenesis in the periovulatory phase and progesterone synthesis during the luteal phase, which has been suggested in prior research^70^ (Table 2).

Fatty acids contained in the phospholipids found reduced in the luteal phase of the present study participate in inflammation modulation through eicosanoid signaling, including linoleic (18:2,ω6), docosahexaenoic (22:6,ω3), stearic (18:0ω6), linolenic (18:3,ω6), arachidonic (20:4,ω6), acids (Table 2). It has been suggested conversion of linoleic to γ-linolenic acid is reduced in PMS^71^. Thus, a state of low linolenic acid could be further augmented in the luteal phase in individuals susceptible to PMS leading to inflammation^72^. PMS symptoms related to inflammation include mood, abdominal cramps, back pain, breast tenderness, appetite cravings, weight gain and bloating^73^. Symptoms of PMS are associated with elevated CRP, which was also observed in the luteal phase^73^; and the elevated luteal phase acylcarnitines may also potentiate a hyperinflammatory state^35^.

Health can be defined as the ability of a living being to adapt and to self manage^74^. The healthy, physiologic state of rhythmicity must be defined to understand the perturbations that need adaptation and management. Identification of biochemical variations in a healthy menstrual cycle can provide a foundation of comparison for future deep phenotyping, such as phenotypic challenges of adaptation^75,76^, for sex hormone related disorders, such as PMS, and PMDD.

This data obtained from healthy women highlights the importance of deeper research on metabolism and sex hormone rhythmicity to understand how therapeutic strategies could be developed to treat challenging medical conditions such as PMS and PMDD. Perturbations in rhythmicity caused by diet, stress and environmental toxins may impact sex hormone related health challenges and result in a loss of rhythmicity and, thus, therapeutic strategies, such as dietary change, may be optimal for restoration. The dietary implications of this study’s findings deserve further testing in a population vulnerable to insufficient diet intake to sustain healthy rhythmicity such as the large population of women with symptoms of PMS and PMDD. Higher protein load, phosphatidylcholine, omega 3^77^ and omega 6^72^ fatty acid intakes may be implicated in the luteal phase along with assurance of sufficient vitamin D intake^65^/sun exposure, B6^78^ sulfur containing vegetables to promote glutathione metabolism^79^, and antioxidant food sources^63^ intake throughout the cycle.

The following summarizes the key limitations in our study. Dietary intake has a significant impact on metabolomics results^80^. While participants limited tea, coffee, fish, alcohol and vigorous exercise 24 h prior to sampling^81^; dietary data were not collected throughout the cycle to assess the associations between differences in food intake and biochemical changes in the blood, plasma and urine. This study was limited in its capacity to detect significant vitamin differences due to missing values from limited sample volumes. In order to develop effective, sensitive diagnostics using metabolomics technologies, more time point measurements could enhance the granularity of conclusions about biochemical changes. Participants were excluded if they were diagnosed with a health condition, however, women who may have had recurrent PMS symptoms in the setting of a healthy menstrual cycle, insufficient to necessitate a medical diagnosis, may not have been excluded.

Our study is, to the best of our knowledge, the first of its kind to conduct a deep phenotyping of the metabolomic, lipidomic, and nutrient biomarker differences across menstrual cycle phases in healthy women. Significant changes in levels of several amino acid and lipid metabolites were identified in addition to those characterized previously^19^. Amino acid and lipid metabolites were reduced in the luteal phase, suggesting differences in anabolic requirements related to changing hormone levels. Rhythmic differences in neurotransmitter related amino acid precursors, vitamin cofactors and stress related metabolites may influence predisposition for anxiety and depression related PMS or PMDD. Glutathione and associated amino acid precursors show rhythmic differences suggesting a greater propensity for oxidative stress throughout the menstrual cycle. The reduction of amino acid levels in the luteal phase combined with prior research on cyclical nitrogen fluctuation, increased energy metabolism and food intake may suggest the intake of a higher protein load as a portion of the increased calorie intake would be advantageous. A menu plan that optimizes protein intake, B6, omega 3 and omega 6 fatty acid and glutathione metabolism deserves further testing in a population at risk of PMS or PMDD. The information generated from this study provides the foundation for research on differences in menstrual cycle, sex hormone related metabolism and clinical biomarker interpretations. Furthermore, it forms the basis to test novel nutrition strategies for women with an emphasis on health issues impacted by rhythmic variability.

## Methods

### Study design

All methods were performed in accordance with the relevant guidelines and regulations. Ethical approval was received from both the Research Ethics Committee, University College Dublin (UCD) and the Commission Cantonale D’Ethique de la Recherche sur L’Etre Humain (CER-VD) in Switzerland. All participants provided written informed consent before study participation. Participants visited the clinic at 4 different timepoints for blood and urine collection at different menstrual cycle phases (Fig. 2). Participants were instructed to keep a menstrual calendar for 1 month prior to sample collection in order to estimate the length of their cycle. Using a guide for menstrual phase length^19^, urine and blood samples were collected from each woman at 4 different time points in one menstrual cycle; estimated to represent 4 stages of the menstrual cycle: menstrual, follicular, luteal and premenstrual (Fig. 1). Participants were instructed to use a luteinizing hormone (LH) urine dip strip test kit (Medimpex Ltd Inc) at home in order to determine the date of ovulation. Following serum hormonal analysis, classification of the phases was refined to define 5 stages of the menstrual cycle (menstrual, follicular, periovulatory, luteal and premenstrual)^19^.

### Study participants

Thirty four healthy, premenopausal women at UCD, Dublin with a mean age of 26.6 years, standard deviation (SD) of +/−5.9; and a mean body mass index (BMI) (Kg/m^3^) of 22.9 +/− 3.5 volunteered to participate in the study. Participants were excluded with a BMI <18 or >30 Kg/m^3^, iron deficiency anemia (hemoglobin <11.5 g/dl), diagnosis with a medical condition and use of prescribed medication or hormonal contraceptives (Fig. 2).

### Blood and urine collection

Prior to blood and urine collection, participants were instructed to fast for 12 h, limit tea and coffee consumption and abstain from fish, alcohol and vigorous exercise for 24 h. On the morning of collection, volunteers collected their first void urine at home in a chilled graduated container and then immediately delivered it to the laboratory on ice. Sample processing and serum hormone analyses were measured at the Biochemistry Department, National Maternity Hospital, Dublin, as previously described^19^.

### Metabolite profiling analysis

Metabolite profiling was done by the Biomedical Metabolomics Facility, Leiden University, Leiden, The Netherlands. All samples (plasma and urine) were randomized and analyzed in batches, which included calibration lines, quality control (QC) samples and blanks. QC samples were prepared from pooled plasma and urine available in the laboratory and were analyzed every 10 samples for data quality and instrument response correction. Blank samples were used to correct for background signal and in-house developed algorithms were applied using the pooled QC samples to compensate for time-dependent drifts of the sensitivity of the mass spectrometer. Data was reported as ratio of analyte signal to internal standard.

The amine platform analyzed 74 amino acids and biogenic amines in plasma and urine using liquid chromatography coupled to a mass spectrometer (LC-MS) employing an AccQ-Tag derivatization strategy adapted from the protocol supplied by Waters (Etten-Leur, The Netherlands)^82^. One μL of the reaction mixture was injected into the ACQUITY UPLC System (Waters, Etten-Leur, The Netherlands) on an AccQ-Tag Ultra column (Waters) for chromatographic separation coupled to a triple quadrupole mass spectrometer (AB SCIEX Qtrap 6500, Framingham, MA USA). Acquired data were evaluated using MultiQuant Software for Quantitative Analysis (AB SCIEX, Version 3.0.2). After quality control correction^83^, 54 plasma amines and 60 urine amines complied with the acceptance criteria of RSDqc <15%.

The lipid platform analyzed 185 compounds in 9 lipid classes in plasma using LC-MS as described^84^. Chromatographic separation was achieved on a ACQUITY UPLC™ (Waters, Etten-Leur, The Netherlands) with a HSS T3 column which was coupled to a ESI-Q-TOF (Agilent 6530, Jose, CA, USA) using reference mass correction^84^. The raw data were pre-processed using Agilent MassHunter Quantitative Analysis software (Agilent, Version B.04.00). After QC and blank correction, 139 compounds comply with the acceptance criteria RSDQC <30% and blank effect <40%. Twenty six organic acids were analyzed in urine by gas chromatography coupled to mass spectrometry (GC-MS). After QC correction and considering blank effects, 23 urinary and 16 plasma organic acids compounds complied with the acceptance criteria RSDQC <30% and blank effect <20%. The plasma and urine metabolites were measured by gas chromatography on an Agilent Technologies 7890A equipped with an Agilent Technologies mass selective detector (MSD 5975C) and MultiPurpose Sampler (MPS, MXY016-02A, Gerstel, Germany). Chromatographic separations were performed on a HP-5MS and detected using a quadrupole mass spectrometer. The raw data were pre-processed using Agilent MassHunter Quantitative Analysis software (Agilent, Version B.05.01).

The endocannabinoid profiling platform analyzed 24 compounds in plasma, as previously described^85^. Chromatographic separation was achieved by an ACQUITY UPLC System (Waters, Etten-Leur, The Netherlands) on an ACQUITY UPLC HSS T3 Column. The UPLC was coupled to electrospray ionization on a triple quadrupole mass spectrometer (AB SCIEX Qtrap 6500, Framingham, MA USA). Acquired data were evaluated using MultiQuant Software for Quantitative Analysis (AB SCIEX, Version 3.0.2). After quality control correction 19 endocannabinoids complied with the acceptance criteria of RSDqc <15%.

The acylcarnitine platform analyzed 45 acylcarnitines as well as trimethylamine-N-oxide, choline, betaine, deoxycarnitine and carnitine in plasma and urine. Chromatographic separation was achieved by UPLC (Agilent 1290, San Jose, CA, USA) on an AccQ-Tag Ultra column (Waters, Etten-Leur, The Netherlands) coupled to electrospray ionization on a triple quadrupole mass spectrometer (Agilent 6460, San Jose, CA, USA). Acquired data were evaluated using Agilent MassHunter Quantitative Analysis software (Agilent, Version B.05.01). After QC correction, 25 urinary and 27 plasma acylcarnitines complied with the acceptance criteria of RSDqc <15%.

The method validation used for the metabolite profiling fits with the acceptance criteria for precision (15% RSD, 20% RSD near LLOQ and accuracy (bias within +/−15% of the accepted reference value, within 20% near LLOQ) specified by the Conference Reports have been widely accepted in bio-analysis^86^. Recommended guidelines were followed for most metabolites, however, some were accepted which were close to the LOQ.

### Clinical and vitamin biochemistry

Standard clinical routine analysis of human serum samples was performed by the Molecular Nutrition laboratory at NIHS. The applied Architect plus ci4100 platform from Abbott Laboratories (Lake Bluff, IL, USA), consists of a chemistry and an immunoassay module^87^. Glucose, insulin, vitamin B12, holotranscobalamin, folate, 25-hydroxy vitamin D and cortisol were determined by chemiluminescent microparticle immunoassays (CMIA) while cholesterol, HDL, triglycerides, high sensitivity C-reactive protein (hsCRP), ceruloplasmin, copper and magnesium were analyzed using the ARCHITECT cSystems assays developed by Abbott Laboratories (Wiesbaden, Germany)^87^.

Plasma analysis was completed by Vitas Analytical Services, Oslo, Norway for B1 (thiamin and thiamin mondphosphate), B2 (FAD, FMN), and B6 (4-pyridoxic acid, pyridoxal 5 phosphate). The analysis was performed with an an Agilent LC-FLD 1200 system using a fluorescence detector (FLD). Separation of the analytes was achieved by a Phenomenex Kinetex® column (2.6 µm C18 100 Å 100 × 4.6 mm). Unknowns were calibrated against known standards from Sigma-Aldrich, and reported as nmol/l.

### Data analysis

Univariate data analysis was used to evaluate changes in metabolite concentrations, clinical, and vitamin data (biochemical species) over five menstrual cycle phases. A mixed model approach was used in which all data was natural log transformed to meet normality assumptions.

In the analysis *Y*_*ijt*_ is the log transformed level of participant *i* in (1, .., 34) for biochemical species *j* in (1, .., 397) at menstrual phase *t* in (1, …, 5). Data were analyzed using the following Linear Mixed Model^88^.

1 $${Y}_{ijt}={b}_{ij}+{\mu }_{jt}+{\varepsilon }_{ijt}$$

The random intercept, ${b}_{ij} \sim N(0,\,{\sigma }_{j}^{b})$, accounts for the dependence of observations from the same participant, *μ*_*jt*_ is the (mean biochemical species *j*) fixed effect in phase *t* and ${{\epsilon }}_{ijt} \sim N(0,\,{\sigma }_{j}^{\varepsilon })$ is an independent, normally distributed error term.

Of interest are pairwise (phase-phase) differences, which we call “contrasts”, defined as $({\mu }_{jt}-{\mu }_{j{t}^{\text{'}}})$ where *t* and *t’* represent two different phases, e.g. luteal and menstrual. The five phases yield ten unique pairwise differences per biochemical species *j*, delivering a total of 3970 (two-sided Student’s t-test) p-values, denoted ${p}_{j}^{c}$ for contrast *c* in (1, …, 10) in biochemical species *j*.

Multiple testing adjustments were made using Bonferroni *within* biochemical species by transforming ${p}_{j}^{c}$ to ${\hat{p}}_{j}^{c}=\,{\rm{\min }}(10\times {p}_{j}^{c},\,1)$, and *across* biochemical species by controlling the false discovery rate contrast-wise using Benjamini-Hochberg (BH)^89^. For each contrast *c* we provide the BH procedure with the ${\hat{p}}_{j}^{c}$ values of all biochemical species from the same panel (amino acids, lipids, et cetera) and sample (plasma, urine, et cetera) resulting in a corresponding set of FDR controlled values ${q}_{j}^{c}$. A pairwise difference *c* for metabolite *j* is considered significantly different from zero if ${q}_{j}^{c} < \,0.20$. All biochemical species with at least one significant contrast are labeled as “entities” and studied further. This threshold is sufficiently liberal to ensure the consecutive pathway analysis is conducted on a rich set of exploratory biochemical species. Result tables are provided so the reader may replicate conclusions using a more stringent threshold if needed (Supplementary Table 2). All statistical analyses were conducted in the R programming language (version R 3.4.0 for Windows) using packages lme4 (1.1–13), lmtest (0.9–35) and multcomp (1.4–6)^90–92^.

### Metabolite reactions and subsystems analyses

The Human Genome Scale Metabolic Model or RECON 2.2.^93–95^ was used for overlaying the metabolite concentration data to map changes in metabolism at different phases to a global/systems scale. Metabolites were input into the RECON model using corresponding KEGG identities^96^. Reactions in the RECON model were identified and filtered for those with meaningful metabolic impact. Impacted metabolic subsystems were then identified based on participating reactions. Interconnectivities and differences between menstrual cycle phases were visualized using custom MATLAB (Mathwork Inc.) scripts and edited using yEd (yWorks GmbH)^17,18,97–99^. (Supplementary Figs 2a,b and 3).

## Electronic supplementary material

Supplementary figure 1

Supplementary tables 1,2
