# Publisher Correction: Menstrual cycle rhythmicity: metabolic patterns in healthy women

**DOI:** 10.1038/s41598-019-41392-x

**Published:** 2019-04-03

**Authors:** C. F. Draper, K. Duisters, B. Weger, A. Chakrabarti, A. C. Harms, L. Brennan, T. Hankemeier, L. Goulet, T. Konz, F. P. Martin, S. Moco, J. van der Greef

**Affiliations:** 1 Nestle Institute of Health Sciences (NIHS), Lausanne, Switzerland; 2 Mathematical Institute, Leiden University, Leiden, The Netherlands; 3 Division of Analytical Biosciences, Leiden Academic Center for Drug Research, Leiden University, Leiden, The Netherlands; 4 Netherlands Metabolomics Centre, Leiden, The Netherlands; 5 University College Dublin, School of Agriculture and Food Science, Belfield, Dublin 4 Ireland

Correction to: *Scientific Reports* 10.1038/s41598-018-32647-0, published online 01 October 2018

In Figure 4, the panels showing the variability by cycle phases for Glycine, Serine, Methionine, Asparagine, Proline, Glutamine, Tyrosine, Gamma-glutamyl-alanine, Citrulline, O-Acetyl-serine, Alpha-aminobutyric acid and Gamma-glutamylglutamine were omitted. The correct Figure 4 appears below as Figure 1.

**Figure 1:**
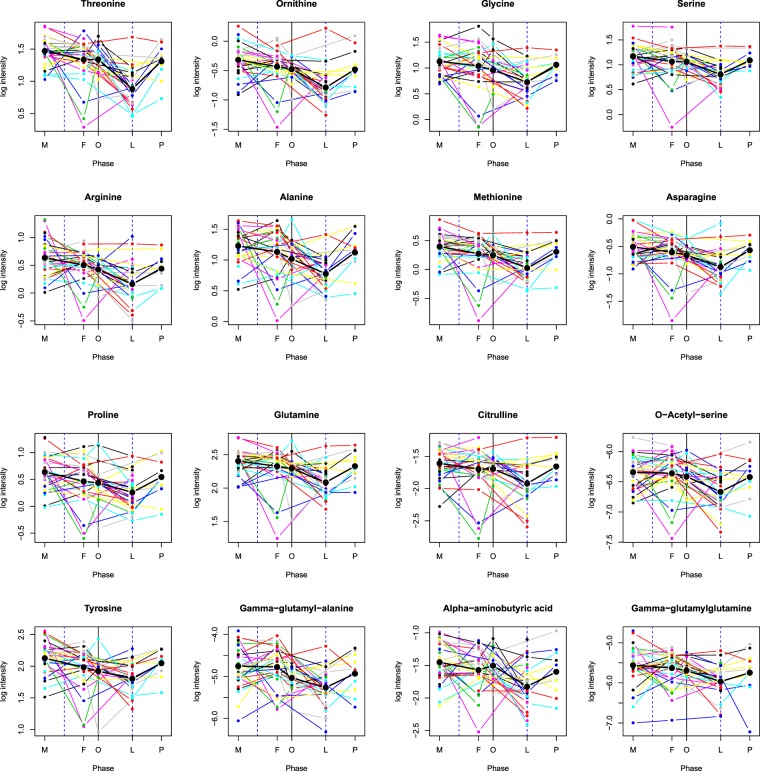
Amino acid variability by cycle phase. Mean log intensity is depicted along with individual variability for threonine, ornithine, arginine, alanine, glycine, serine, methionine, asparagine, proline, glutamine, tyrosine, gamma-glutamyl-alanine, citrulline, o-acetyl-serine, alpha-aminobutyric acid, and gamma-glutamylglutamine at one time point for each of the 5 menstrual phases (M = menstrual, F = follicular, O = periovular, L = luteal, p = premenstrual). Each colored line represents an individual. Amino acids are depicted which have 2 or more contrast comparisons meeting the multiple testing threshold of q < 0.20. Statistically significant luteal phase reductions can be observed.

