# Supplementary material for: Menstrual cycle rhythmicity: metabolic patterns in healthy women
Source: Sci Rep. 2018 Oct 1;8:14568. doi: 10.1038/s41598-018-32647-0 (PMC6167362; doi:10.1038/s41598-018-32647-0)
Supplement: Supplementary file 1 — Supplementary figure 1 [file 41598_2018_32647_MOESM1_ESM.pdf]

Supplementary Figures
Menstrual cycle metabolic rhythmicity: biomarkers of health

Draper CF[1], Duisters K[2], Weger B[1], Chakrabarti A[1], Harms AC[3], Brennan L[4], Hankemeier T[3], Goulet L[1], Konz T[1], Martin FP[1], Moco S*[1], van der Greef J*[3]

1 Nestle Institute of Health Sciences (NIHS), Lausanne, Switzerland
2 Mathematical Institute, Leiden University, Leiden, The Netherlands
3 Division of Analytical Biosciences, Leiden Academic Center for Drug Research, Leiden University, Leiden, The Netherlands; Netherlands Metabolomics Centre, Leiden, The Netherlands
4 University College Dublin, School of Agriculture and Food Science

*Shared senior authors

Correspondence to: colleen.draper@rd.nestle.com Tel.: +41 21 632-6154

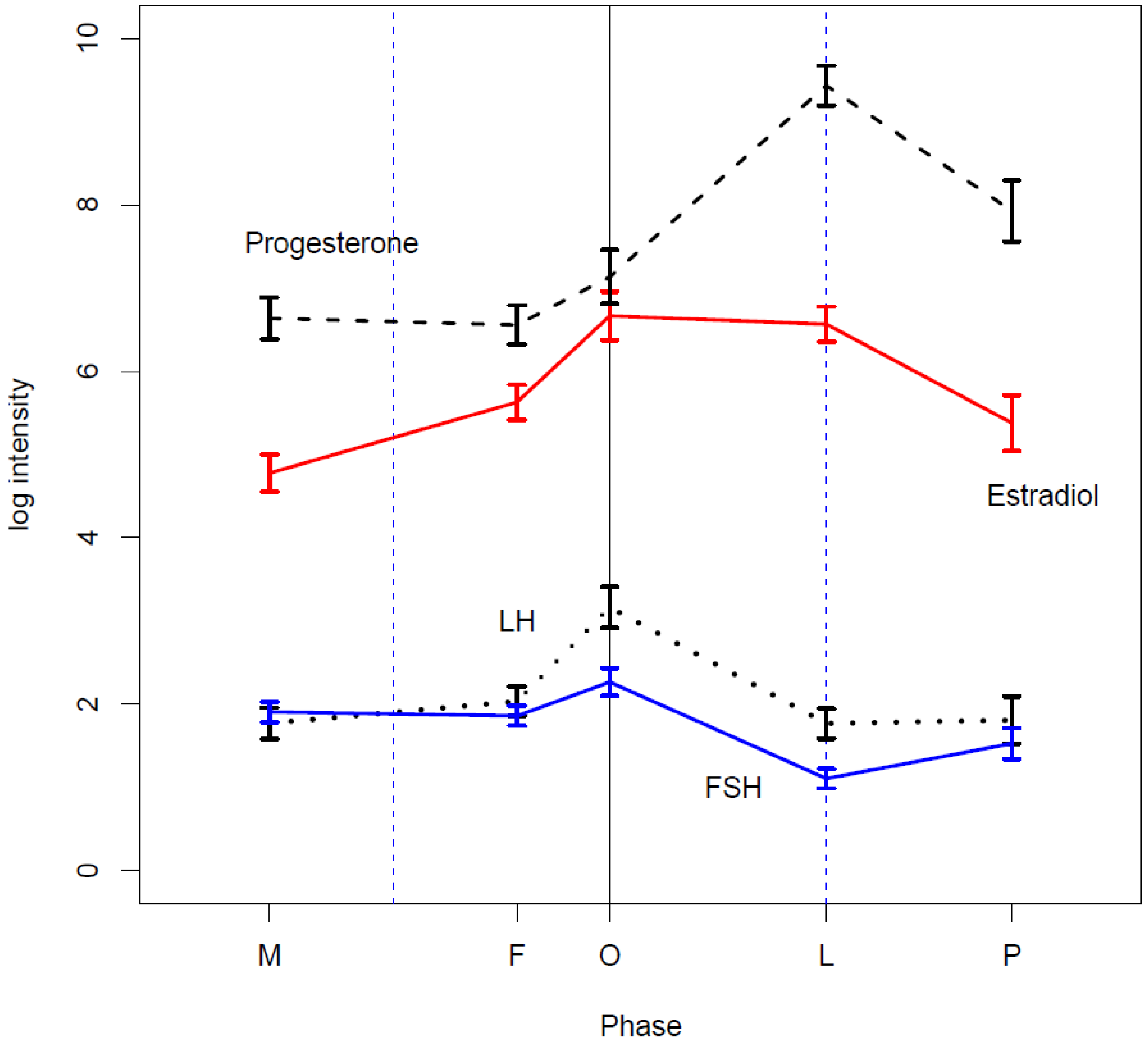

Supplementary Figure 1. Sex hormones per cycle phase

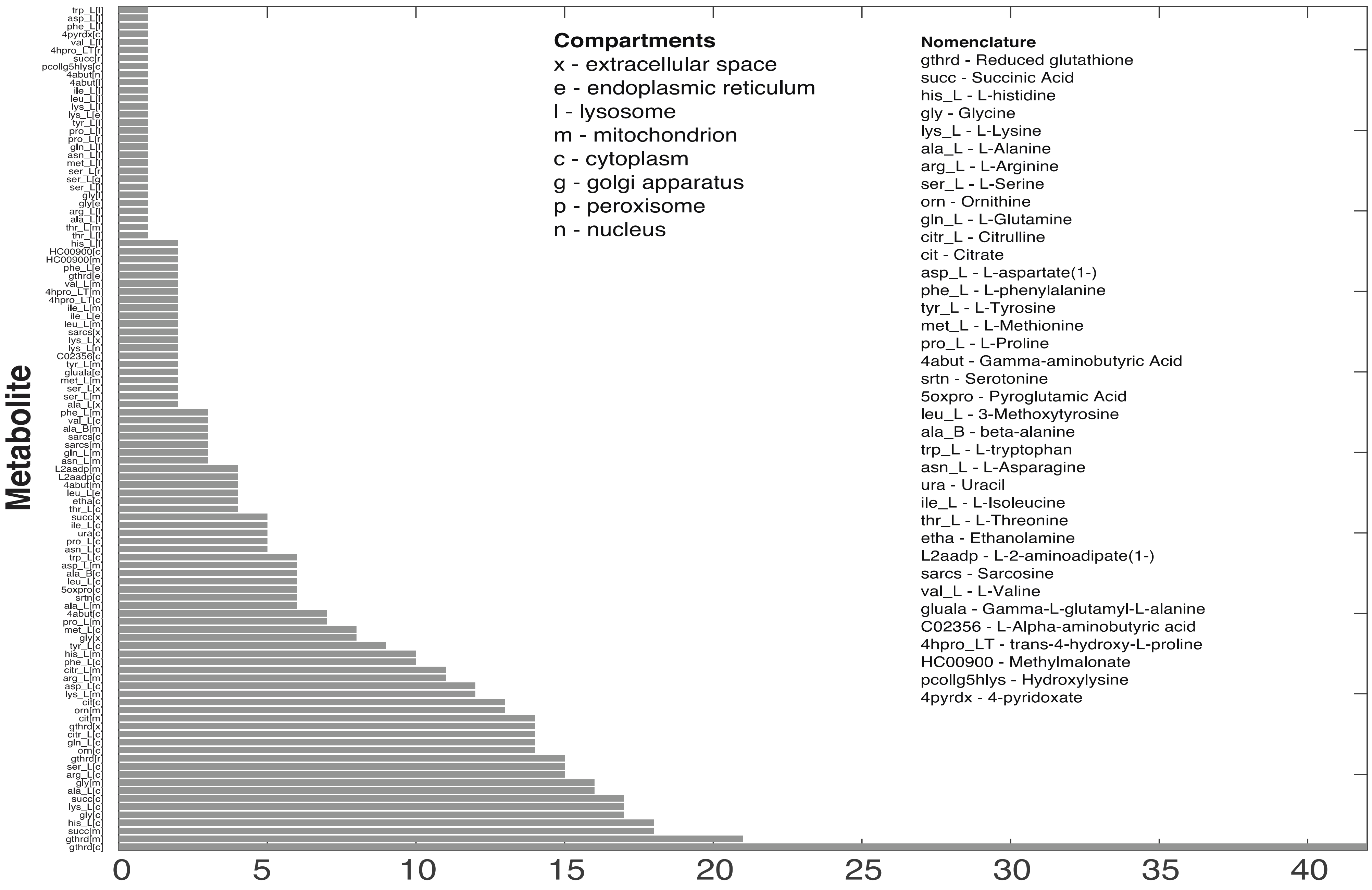

Supplementary Figure 2a. Reactions per metabolite

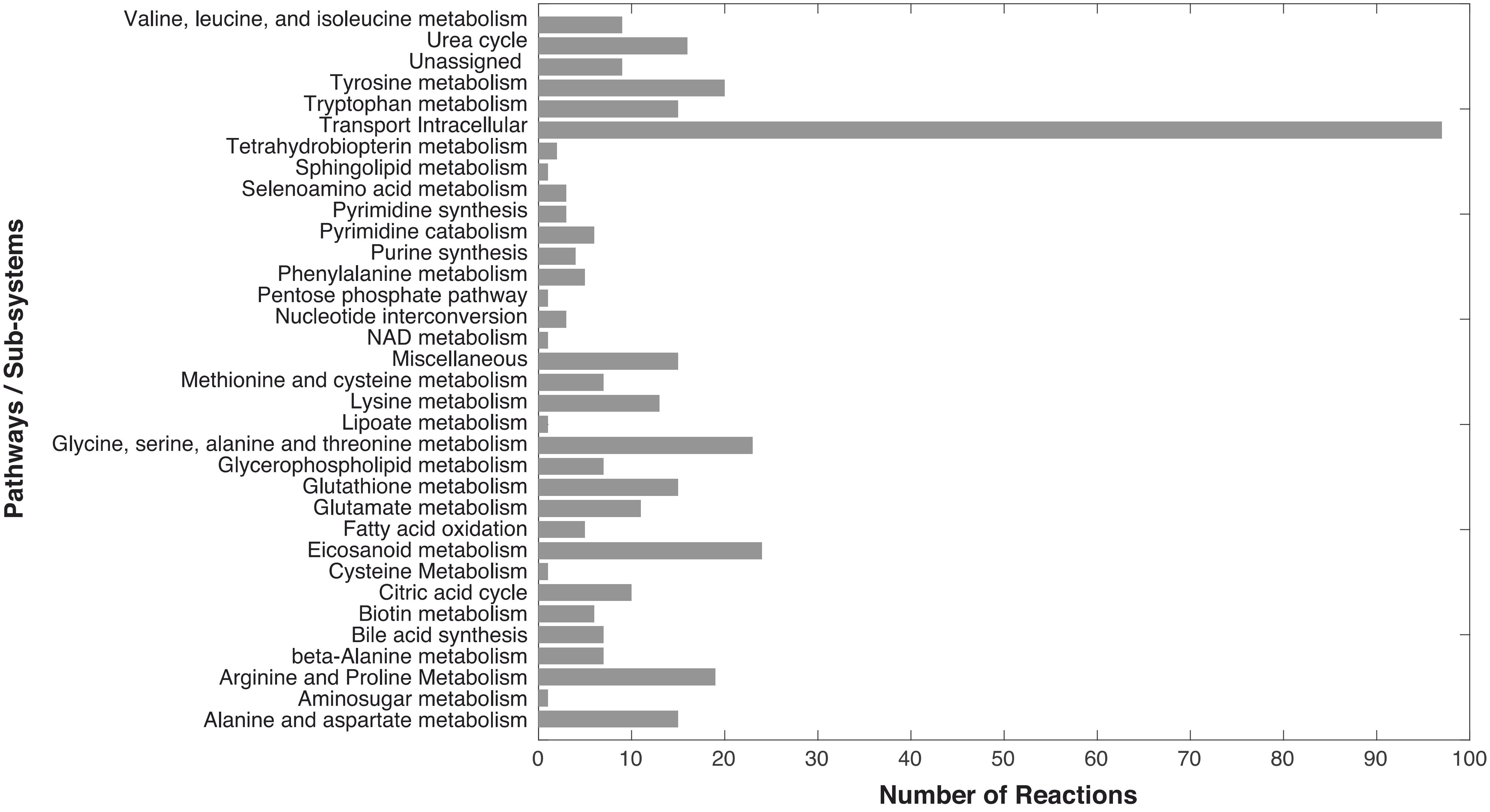

Supplementary Figure 2b. Reactions per impacted sub-system

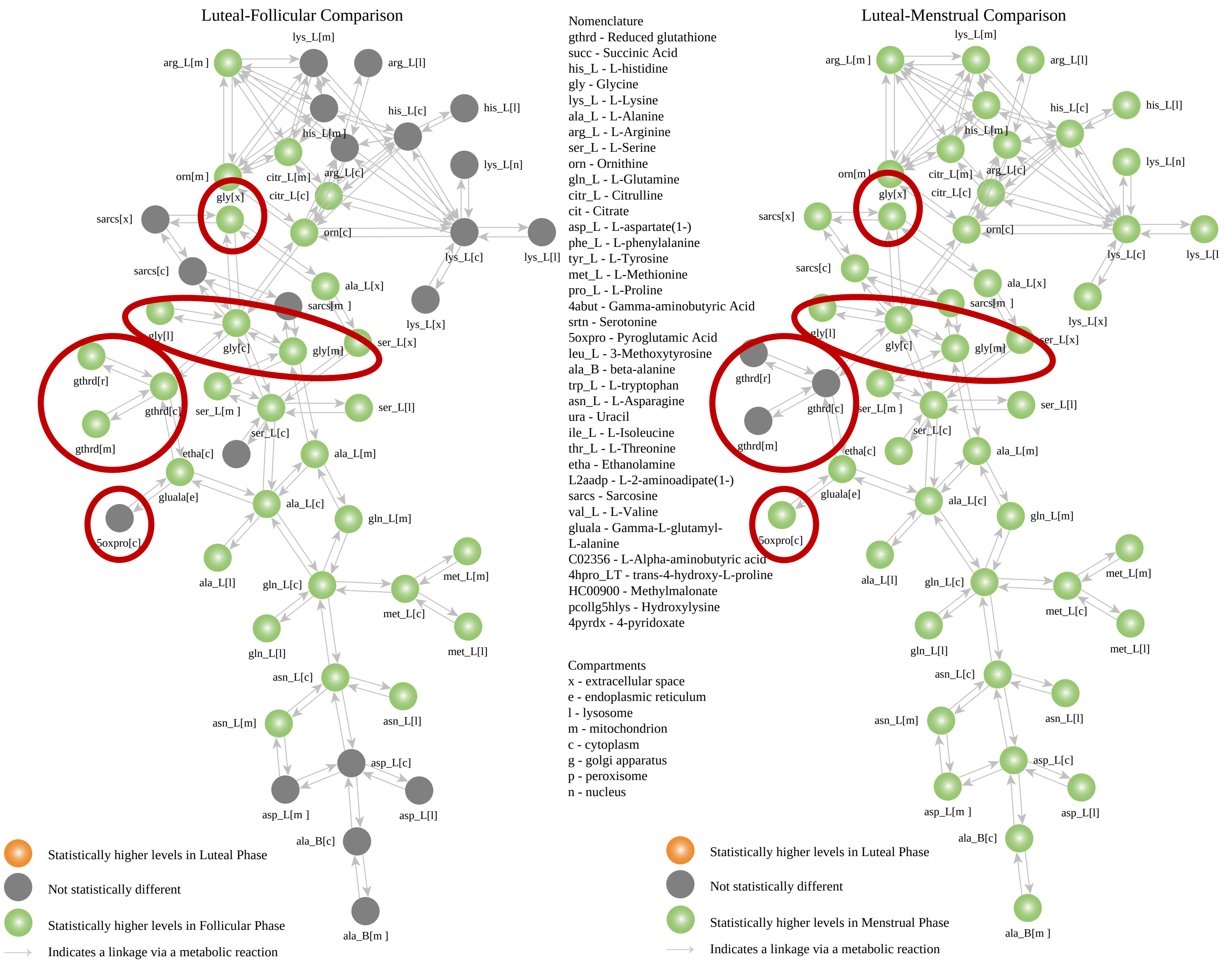

Supplementary figure 3. Inter-connected metabolites reduced in luteal to follicular and luteal to menstrual contrasts

Supplementary Fig. 1. Sex hormones per cycle phase.
Illustrates the concentration of 4 sex hormone levels across phases in the current study. Serum hormone levels, urinary luteninizing hormone (LH) for date of ovulation and self-reported menstrual cycle length were used to determine menstrual cycle phase. FSH = follicular stimulating hormone; M = menstrual; F = follicular; O = peri-ovulatory; L = luteal; P = premenstrual phases.

Supplementary Fig. 2a. Reactions per metabolite and impacted sub-systems.
The connectivity of the 41 metabolic entities within the 419 reactions was analyzed using the Human Genome Scale Metabolic Model or RECON 2.2. Reduced glutathione, succinate, L-histidine and glycine followed by L-lysine, L-alanine, L-arginine and L-serine are impacting/impacted by the most number of reactions.

Supplementary Fig. 2b – Thirty four subsystems which comprise the 419 reactions were identified within the metabolic landscape using the Human Genome Scale Metabolic Model or RECON 2.2. The spread across different pathways is shown. Affected sub-systems include amino acid metabolism/synthesis, the urea cycle, eicosanoid metabolism, citric acid cycle and bile acid synthesis.

Supplementary Fig. 3. Interconnected metabolites (L-F, L-M).
A metabolite connectivity network is illustrated to assess how the differentially observed metabolites are dependent on each other and accordingly express an interdependent change. Any metabolite is connected to another metabolite, if they are participating in a reaction, i.e. they are either products/reactants and vice versa. Interconnectivities and differences between menstrual cycle phases were visualized using custom MATLAB (Mathwork Inc.) scripts and edited using yEd (yWorks GmbH). To visualize and analyze the interconnections within the reactome, any line with an arrow denotes a direct relationship between the two reactions. The maximum number of differences are observed between the menstrual and the luteal phase, with the key set of metabolites methionine, asparagine, b-alanine, glutamine, alanine, serine, glycine, ornithine, arginine and lysine forming the core backbone of interconnected metabolites impacted by Luteal-Follicular and Luteal-Menstrual phase differences. Red circles are used to denote glutathione and its' related precursors.
